# Prognostic Factors for Hearing Preservation Surgery in Small Vestibular Schwannoma

**DOI:** 10.3390/audiolres13040042

**Published:** 2023-07-03

**Authors:** Stefano Concheri, Alessandra Deretti, Giulia Tealdo, Elisabetta Zanoletti

**Affiliations:** Section of Otorhinolaryngology-Head and Neck Surgery, Department of Neurosciences, University of Padova, 35128 Padova, Italy

**Keywords:** vestibular schwannoma, acoustic neuroma, hearing preservation surgery, prognostic factors, retrosigmoid approach, middle fossa approach, preoperative hearing, tumor size, ABR

## Abstract

Objective: to evaluate recent contributions to the literature on prognostic factors of hearing preservation in small vestibular schwannoma microsurgery. Methods: review of the most recent studies. Results: factors such as tumor size, preoperative hearing status, tumor growth rate, tumor origin, surgical approach, radiological characteristics, results of preoperative neurophysiological tests, preoperative symptoms and demographic features have been investigated and some of them reported to be significant in the prediction of hearing preservation. Conclusions: tumor size and preoperative hearing status are the most impactful factors and play a key role in patient selection for hearing preservation surgery. Other features such as fundal extension, tumor origin and impaired ABR could have prognostic value on hearing preservation. Tumor growth rate, preoperative impedance, cVEMPs and age have also recently been found to be significant, but more studies are needed. The role of preoperative tinnitus, vertigo and gender is lacking and controversial, whereas the differences between available surgical approaches have been smoothed out in recent years.

## 1. Introduction

Vestibular schwannoma (VS) is a benign neoplasm, usually arising from the vestibular branch of the eighth cranial nerve, but different origins are possible. It represents the most common lesion of the cerebello-pontine angle (CPA) and 8% of all intracranial tumors [1].

Its real incidence is still debated and can vary from 1 to 20 cases per million, according to different studies [2], but most recent works report 1–2 cases per 100,000 inhabitants, with a possible higher level of diagnosis in the European population [3]. Since the 1970s, there has been a gradual raise in incidence, with a peak in diagnosis in 2003–2005, followed by a progressive decrease to the actual value [3,4,5]. This trend has been explained by increased use of imaging, increased attention to oto-vestibular symptoms, and increased life expectancy, rather than a real increase in tumor incidence [5].

Despite a potentially long asymptomatic phase, one or more of the following symptoms, such as hearing loss, tinnitus, and vestibular dysfunction, are a common finding in VSs patients. Typically, the otological clinical examination is completely negative, while pure tone audiometry may show a unilateral sensorineural hearing loss (SNHL) or an interaural difference in hearing threshold. The vestibular clinical examination may be impaired, but there are no pathognomonic findings. The videonystagmography (VNG) is more sensitive in identifying vestibular nerve disfunction and may also quantify the damaged number of fibers. Neurophysiological tests (e.g., auditory brainstem response—ABR) are used to determine the damage on the cochlear nerve. The facial nerve is often unaffected by small tumors, while larger tumors may present with subclinical or clinical nerve deficit; this can be assessed using the House-Backmann scale and quantified with electromyography.

When a VS is suspected, high-resolution contrast-enhanced MRI is the gold standard to confirm diagnosis. T1 sequences enable identification of a mass in the CPA, iso-hypointense to the cerebral tissue, with intense gadolinium-contrast enhancement; when evaluated in T2, the tumor appears hyperintense. CSF-cisternographies sequences (e.g., CISS/FIESTA seq.) are useful for visualizing cranial nerves and vascular structures at the skull base-sequences and allow accurate for evaluations of tumor relations with them; these sequences are also essential to determining VS extension to the fundus of the internal auditory canal (IAC). High resolution CT scans are useful for evaluating petrosus bone structures in a preoperative setting; they also allow comparisons with the size of the affected IAC, which can be enlarged by the tumor, with the contralateral IAC.

The optimal way to calculate tumor dimension was initially discussed in the first Acoustic Neuroma Conference in 1991 (Copenhagen), but no clear agreement was reached. In 2001, forty specialists met in Tokyo in the Consensus Meeting on Systems for Reporting Results in Acoustic Neuroma [6]; the linear tumor size of the largest available extrameatal diameter was assessed in gadolinium-enhanced axial T1 sequences and “the intracanalicular or intrameatal and extrameatal portion of the tumor should be clearly distinguished”; the length of the intrameatal portion should not be added to the extrameatal tumor diameter. Classification of VSs according to their dimension was defined as reported in Table 1; a small VS is between 1 and 10 mm in diameter [6]. The classification proposed by Koos in 1998 considers tumor relation with the IAC, the CPA and the brainstem and divides VSs in four grades (I–IV) [7].

In the last decades, the increasingly widespread and early use of MRI to assess otological and neurologic symptoms has led to an increased discovery of small tumors in the initial phase of their natural history. These patients usually have no complaints, and their hearing function is often unaffected, making it necessary to implement treatment options that preserve hearing function.

The most widely used scales to report hearing outcomes are Tokyo [6] and AAOHNS [8], both based on PTA2 (Pure Tone Average at 500 Hz, 1000 Hz, 2000 Hz and 3000 Hz in the AAOHNS or 4000 Hz in Tokyo) and SDS values (peak percentage of speech discrimination). The Tokyo scale considers 6 hearing classes (A–F); the second, adopted in 1995, considers only 4 (A–D). The Gardner-Robertson [9] scale is also available.

To date, treatment of small tumors is still a debatable issue and is the object of the present review.

The available therapeutic strategies are: observation with periodic MRI scan, microsurgery, or radiosurgery. Small tumors are traditionally proposed for observation at first, with annual repetition of the MRI scan; active treatments are reserved for growing tumors. However, it has been recently shown that early surgery in small tumors can lead to clear advantages for the patient, in terms of curing disease, hearing function and preservation of the facial nerve, with better outcomes than conservative treatments [10,11]. The role of hearing preservation surgery (HPS) becomes especially evident in the long-term follow up [10,11,12,13]. Among the available surgical procedures, the retrosigmoid approach (RS), with retrolabyrinthine meatotomy (RLM) [11,13] or, in selected cases, endoscopic-assisted, is currently the most used method for removing the tumor from the internal auditory canal in HPS. In our experience [13], this technique allows for the complete removal of the tumor, providing a considerable rate of hearing function preservation (71% A-B classes of Tokyo classification and 85% A-B classes of AAOHNS) [13] and an excellent facial nerve outcome [13]. Other hearing-preservation approaches are the middle cranial fossa approach (MCF), for purely intrameatal tumors, and the retrolabyrinthine-presigmoid approach, for selected small tumors in the CPA, where the combined use of the endoscope helps to remove a limited extension of the tumor in the internal auditory canal. The key issue in HPS is the preoperative selection of patients. Tumor size and hearing play a crucial role, in combination with the patient’s age and adequate counseling about the possible therapeutic alternatives. In our center, HPS is considered a convenient option in <10 mm tumors in the CPA and hearing function with PTA2 < 30 and SDS > 70%, i.e., class AB and class A of Tokyo and AAHONS, respectively [11].

However, the role of the factors influencing and predicting the hearing outcome in the preoperative setting is still debated. This has promoted investigation of possible prognostic factors that would help to select the best candidates for HPS in relation to the inclusion criteria, with the aim of improving the outcome through the identification of reliable prognostic predictors on the likelihood of retaining hearing [14] when using the HPS strategy.

This review focuses on the most recent contributions investigating which prognostic factors can predict successful outcomes, with the aim of selecting the best candidates for HPS.

## 2. Materials and Methods

For this narrative review, the most recent studies published on hearing preservation in the surgical management of vestibular schwannoma were considered. The research conducted through the Pubmed library using the proper MeSH term for VS (“Neuroma, Acoustic” [Mesh]), combined with other terms referring to hearing preservation surgery (“hearing preservation surgery”, “HPS”, “hearing preservation”, “retrosigmoid” approach, “middle cranial fossa” approach); both RS and MCF approaches were included. Duplicate records were removed, and the remaining ones were screened for suitable records, i.e., studies investigating prognostic factors for HPS in LV patients. Papers not in English or unavailable for consultation were excluded.

No mandatory time interval has been set a priori in order to obtain as comprehensive a collection of factors as possible; on the other hand, when a large amount of literature was available, only the most updated works were considered; in some cases, older studies were also selected to allow for discussion of the changing role of prognostic aspects over the years.

When the literature investigating the role of a certain factor in the outcomes of HPS in small tumors was available, case series referring to the same predictor in larger VSs were excluded, since the group of small tumors represents the target of the hearing preservation procedures.

## 3. Results

The reported prognostic factors are tumor size, growth rate, preoperative hearing status, vestibular nerve of origin, surgical approach, presence or absence of the “fundal fluid cap” and other radiologic characteristics (FLAIR signal). Other preoperative neurophysiological tests (ABR, impedance and cVEMPs), symptoms (tinnitus, vertigo) and demographic features (age, gender) were investigated, too.

Articles selected for this review are shown in Table 2. The oldest dates back to 1990, and the most recent to 2022; 65% of the studies were published after 2010.

The considered prognostic factors are summarized in Table 3, which reports whether these factors were found to be significantly prognostic or not, that is, to be statistically associated with better hearing results thanks to appropriate statistical tests and a *p*-value < 0.05.

## 4. Discussion

### 4.1. Tumor Size

Tumor size is the first identified and one of the best-known factors that plays a leading prognostic role and greatly influences patients’ selection.

This aspect was initially investigated by Kemink and colleagues in 1990, who identified a size <1.5 cm as a criterion for predicting good postoperative hearing outcomes [15]. The role of tumor size was subsequently confirmed by various studies, although there are also studies in the literature that have not found statistical significance [16,17]. The paper with the largest number of patients was conducted by Robinette and Mohr, who analyzed, respectively, 104 and 128 cases that had undergone HPS via the RS approach. Both authors agreed in considering an increased tumor size as a factor related to unsuccessful hearing outcomes [18]; additionally, Mohr calculated a cutoff of 15 mm or smaller as independent favorable factors in hearing preservation (OR 37.77) [19]. In 2020, Ren’s study on 151 patients confirmed that smaller tumor size provides better chances for sparing hearing function (OR of tumor diameter: 0.892); best results are achieved in intracanalicular tumors [20].

Gjurik et al. tried to correlate both tumor volume and linear dimension with postoperative audiological outcomes; in their study, only patients with a lesion volume <0.20 cm^3^ were considered to have significantly better chances for preserving hearing function, while tumor dimension assessed with linear measurement did not reach statistical significance [16].

In 2020, Zanoletti et al., retrospectively analyzed hearing outcomes in a cohort of consecutive patients (100 cases) treated with a HPS procedure. They all were diagnosed with VS and operated on between 2000 and 2012 using the microscopic retrosigmoid approach combined with retrolabyrinthine meatotomy; the multivariate analysis concluded that an extrameatal tumor size of less than 7 mm was an independent predictor of good postoperative hearing function (sensitivity 83.8, specificity 51.8, negative likelihood ratio 1.742, positive likelihood ratio 0.311) [14].

Based on the reported literature and our experience [11,13,21], we propose HPS only to selected patients with a small VS, i.e., <10 mm in the CPA, measured according to Modified Reporting Systems from the Consensus Meeting on Systems for Reporting Results in Vestibular Schwannoma [6].

### 4.2. Tumor Growth Rate

A recent paper by Lovato et al. has retrospectively analyzed 92 patients with a small-medium tumor who underwent an operation using the MCF approach between 1995 and 2015. A tumor growth rate >2.16 mm/year was found to be an independent negative predictor for successful HPS via MCF (OR 8.5) [17]. Further studies are needed to adequately understand the role of this factor in predicting good hearing preservation, and to evaluate if it is reliable in HPS via the RS approach.

### 4.3. Preoperative Hearing

Pure tone audiometry and speech audiometry in VS patients are the most used tests for assessing preoperative hearing status and estimating tumor damage on the cochlear nerve. The prognostic role of preoperative hearing function in relation to the postoperative hearing preservation rate has been widely debated. Although useful to group results, the use of different classification systems (AAO-HNS [8], Tokyo [6] and Gardner-Robertson [9]) risk greatly reducing our ability to compare hearing outcomes [14].

A recent study by Ochal-Choińska and colleagues analyzed audiological parameters with a prognostic value in predicting good hearing preservation after surgery; a positive correlation was found for ipsilateral hearing thresholds at 125 Hz, 500 Hz and 1000 Hz and for PTA2. A speech discrimination score (SDS) for individual sound intensities from 55 dB to 75 dB in the operated ear and speech detection threshold (SDT) were found to correlate with good hearing outcomes, but no significance was obtained for the SDS value [22]. Di Maio et al. retrospectively analyzed a cohort of 192 large VSs operated using the RS approach and found a positive association between high preoperative hearing class and successful HP [23]. The same results were obtained by Lovato et al. in 92 cases operated via the MCF approach: patients with hearing class A or B according to AAO-HNS achieved better postoperative hearing outcomes (OR 5.89) [17]. The 138 cases operated on by Huo et colleagues (RS and MCF) were more likely to achieve hearing preservation after surgery if they had useful hearing before the operation (Class A or B, according to AAO-HNS; OR 0.204); the considered VSs were small and medium-sized (<20 mm) tumors and never larger than grade 2 according to the Koos Classification system [24].

Our cohort of patients were treated using a RS approach and retrolabyrinthine meatotomy, and the results were recently published; the median tumor size was 10 mm in the CPA. Statistical analysis showed that the optimal preoperative hearing threshold cutoffs for hearing preservation purposes are 21 dB PTA (sensitivity 74.2, specificity 66.7, negative likelihood ratio 2.226, positive likelihood ratio 0.387) and 90% SDS (sensitivity 75.4, specificity 44.4, negative likelihood ratio 1.357, positive likelihood ratio 0.553) and confirmed that better preoperative hearing status allowed for lower rates of post-surgical hearing loss. This can be explained by the greater reserve of functional fibers present in patients with good hearing, which would therefore better support the surgical damage [14]. To date, patients with preoperative hearing class A (AAOHNS) or A-B (Tokyo), i.e., a ≤30 dB PTA2 and ≥70% SDS, were considered good candidates for HPS.

### 4.4. Tumor Origin

Schwannomas of the pontocerebellar angle usually arise from the vestibular branch of the 8th cranial nerve, but other origins are reported, i.e., the cochlear nerve or facial nerve [25]; at the fundus of the internal auditory canal, the vestibular nerve divides into the superior (SVN) and inferior vestibular nerve (IVN). The most frequent subseat originating the tumor is not clear: it appears to be the upper one in recent studies (57.6–59.2%) [24,26], but an equal distribution or a major percentage of IVN origin are also reported [25,27,28]. Preoperatively, the involved branch may be successfully estimated using a combination of audiological tests, such as VEMPs and the caloric test [26]. In literature, the removal of IVN tumors achieves a significantly higher ratio of impaired hearing compared to SVN tumors (Huo OR 4.570) [24,27,28]. This may be explained by the proximity of the IVN to the cochlear nerve, so that the dissection may damage the auditory nerve itself, or its blood supply [25].

### 4.5. Surgical Approach

Retrosigmoid (RS) and middle cranial fossa (MCF) are the most used surgical approaches for hearing preservation in VS surgery. The difference between hearing outcomes provided by these techniques has been investigated since the end of the twentieth century; early research seems to show a higher prevalence of hearing preservation in MCF compared to RS [29], respectively 63% vs. 47% [30]; this difference was more evident for tumors <1.5 cm [31]. In the following years, studies showed a progressive improvement in hearing results with the RS approach (even if results vary a lot between different centers due to the heterogeneous nature of surgery itself), and the difference with MCF started to become statistically insignificant [32,33].

Most recent literature seems to provide an inversion in hearing outcomes, with comparable or better ratio of preservation provided by the RS approach (OR 0.744) [24,34], although without reaching statistical significance. This is probably due to the greater confidence among the oto-neurosurgical community in this approach, and to the diffusion of the retrolabyrinthine meatotomy [35]. On the other hand, MCF has been correlated with a high rate of facial nerve impairment [31,33]. In our center, RS with retrolabyrinthine meatotomy is the preferred approach for hearing preservation procedures in small VSs with good preoperative hearing. Auditory and facial nerve outcomes are favorable [13].

### 4.6. Radiological Characteristics

VSs are preoperatively evaluated with gadolinium enhanced MRI. High resolution T2 sequences (cerebrospinal fluid—cisternography sequences) allows assessment of the presence of the “fundal fluid cap”, i.e., the hyperintense signal corresponding to the presence of cerebrospinal fluid (CSF) at the fundus of the internal auditory channel (IAC). This is typical in VSs not involving the lateral part of the canal. The correlation between this aspect and HP was investigated in literature and results were initially ambiguous. In 2003, Kokaoglu retrospectively studied 22 patients, operated on using either the RS or MCF approach, and confirmed that a tumor involving the fundus or a short distance between the tumor and the fundus are risk factors for unsuccessful HPS [36]. The positive predicting role of the fluid cap was confirmed by the study of Goddard in relation to the MCF approach [27]. Otherwise, Sun et al. recently reviewed their 138 MCF cases and registered the presence of a fundal fluid cap; statistical analysis did not find a significant correlation between increasing fundal fluid size and low postoperative PTA or a high word recognition score (WRS) [37]. Afterwards, large case studies of the RS approach have been described by Tringali et al., in 2010 and Nguyen et al. in 2012, both of which confirmed the lateral extension of the tumor to the fundus as a negative predictive factor for HPS [38,39]. In our experience, a fundus-involving tumor is not necessarily an unfavorable condition for HPS via RS, although the compression of the nerve in this site may severely damage the fibers of the cochlear nerve, and the intraoperative dissection in the fundus may endanger the cochlear nerve preservation; on the other hand, the retrolabyrinthine meatotomy allows complete exposure of the whole internal auditory canal and a safe exposure of the fundus which reduces the at-risk surgical maneuvers of dissection at the fundus, with safe handling of the tumor-nerve. Preoperative MRI findings may also not be predictive of intraoperative conditions [40].

In 2019 a study by Tu et al. evaluated the preoperative and postoperative cochlear signal on FLAIR sequences in VSs cases which had undergone HPS with RS or MCF approaches. Preoperative signal does not change between preserved and unpreserved HPS, but postoperative values are lower in successful cases [41].

### 4.7. Preoperative Neurophysiological Testing (ABR, Impedance and cVEMPs)

Preoperative assessment of VS patients should also involve neurophysiological tests to better evaluate tumor damage on the nerve. Auditory brainstem response (ABR) is the most used, due to its easy execution and low costs. Many papers have studied the relation between this test and hearing outcomes without finding any correlation [18,42,43]; nevertheless, its role is so important that in our experience an absent preoperative ABR is considered a contraindication to HPS. In 2019, Ochal-Choińska and colleagues analyzed their series of 86 patients (all operated on using the MCF approach) to determinate whether and which ABR findings could be considered a prognostic factor for postoperative hearing function; they found that presence of wave V, lower values of I–V and III–V intervals and higher amplitude value of wave V in the operation correlates with better HPS results [22]. Our recent analysis of 57 patients who had undergone resection via the RS approach confirmed the role of an absent or severely impaired preoperative ABR as a negative prognostic factor for hearing impairment after HPS (OR 13.38 according to AAO-HNS and 8.1 to Tokyo) [14].

Preoperative impedance was also evaluated and successfully correlated with postoperative hearing outcomes: intensity level needed for simulating the stapedial reflex and abnormal reflex are recently reported as prognostic factors [22].

In 2018, Hochet suggested that cVEMPs may be useful for predicting HP outcomes; they calculated the preoperative amplitude asymmetry ratio in their VS patients: it was significatively lower in the group with successful HP compared with the other one. They calculate that an amplitude asymmetry less than 24% has a positive predictive value for assessing postoperative hearing preservation of 91.6% [44].

### 4.8. Symptoms Other than SNHL

Vestibular schwannoma patients may be asymptomatic or suffer mainly from sensorineural hearing loss, tinnitus and/or vertigo; while the role of the former as an unfavorable predictor of HP has been widely described [14,22,23,24], the others have not been adequately investigated. The literature is lacking studies on the predictive role of vestibular disorders on HP. According to a study conducted in 1995, patients who suffer from vertigo do not differ statistically in HP outcomes compared with those who do not complain of vestibular disorder [45].

Preoperative tinnitus has recently been proposed as an indicator of poor hearing reserve and appears to predict low HP rate [46], but more studies are necessary.

### 4.9. Demographic Features

Age and gender are demographic features whose role was investigated as prognostic factors for HP. The first one has been reported in the past without reaching statistical significance [42,43], but a recent study by Ren et. colleagues confirmed a higher rate of HP in younger patients [20], confirming the role of age hypothesized by Sughrue et al. in 2010 [30].

Gender has occasionally been evaluated, e.g., by Nadol et colleagues, without finding any difference between male and female patients [47]. Ultimately, a recent study conducted at our institution evaluating these aspects found no role in predicting HP outcomes [14].

### 4.10. Other Factors

In addition to the varyingly impactful roles of the factors examined in this review, other aspects may influence the audiological outcomes in hearing preservation surgery in VSs and need to be mentioned. As in every surgical procedure, the surgeon’s experience has a certain impact in the outcome; in particular, hearing preservation in VSs needs a very delicate dissection of the cochlear nerve so as to not damage the fibers and or reduce its blood supply. Such complicated procedures should be performed in a tertiary referral center by well-trained surgeons.

Moreover, different techniques have been proposed to monitor the nerve during surgery, i.e., Auditory Brainstem Responses (ABR), Direct Eighth Cranial Nerve Monitoring, Cochlear Compound Nerve Action Potentials (CNAP), Transtympanic Electrocochleography, Distortion Product Otoacoustic Emissions, Postauricular Muscle Responses. All of them may play a role in hearing preservation, but literature is lacking standardized studies, necessary for evaluating their true efficacy, determine the best one for each situation and understand their predictive role for HPS, as a recent review concluded [48]. To date, the choice of the correct method for monitoring the cochlear nerve is left to surgeons and should be based on the characteristics of the tumor, the patient, and the surgery [48].

## 5. Conclusions

The most important factors showing a predictive role in hearing preservation are tumor size and preoperative hearing status; these features play a prominent role in the selection of patients undergoing HPS. Other reported relevant features which might influence the postoperative outcome, although without strong statistical evidence, are fundal extension, tumor origin and, as evidenced in our experience too, impaired ABR. Recently, tumor growth rate, age, preoperative impedance and cVEMPs have also been debated. Studies evaluating the prognostic role of preoperative symptoms (except hearing loss) and gender are lacking. The differences between surgical approaches (the middle cranial fossa and retrosigmoid approach) have smoothed out in recent years and are more greatly influenced by the experience of the surgeon than by objective factors; the combination of the retrolabyrinthine meatotomy with the RS approach to fully expose the whole IAC, on the other hand, has provided safer handling of the tumor-nerve interface at the distal end of the meatus. The role of intraoperative cochlear nerve monitoring techniques has yet to be properly investigated.

## Figures and Tables

**Table 1:** Tumor classification based on extrameatal dimension, according to the Consensus Meeting on Systems for Reporting Results in Acoustic Neuroma (Tokyo, 2001).

1–10 mm	Small	Grade 1
11–20 mm	Medium	Grade 2
21–30 mm	Moderately large	Grade 3
31–40 mm	Large	Grade 4
>40 mm	Giant	Grade 5

**Table 2:** Studies considered for this review.

First Author	Year	Design	Patients (n)
Kemink	1990	RCS	20
Fischer	1992	RCS	99
Nadol	1992	RCS	144
Cohen	1993	RCS	161
Robinette	1997	RCS	104
Selesnick	2001	RCS	6
Kocaoglu	2003	PCS	22
Moh	2005	RCS	386
Gjuric	2007	RCS	29
Jacob	2007	RCS	356
Noudel	2009	NR	n.a.
Tringali	2010	RCS	278
Goddard	2010	RCS	101
Sughrue	2010	NR	998
Sameshima	2010	RCS	504
Rachinger	2011	RCS	90
Mazzoni	2011	RCS	115
Di Maio	2011	RCS	192
Nguyen	2012	RCS	53
Ansari	2012	NR	n.a.
De Freitas	2012	RCS	175
Mazzoni	2018	RCS	100
Sun	2018	RCS	138
Hochet	2018	RCS	18
Lovato	2019	RCS	92
Tu	2019	RCS	18
Ochal-choińska	2019	RCS	86
Huo	2019	RCS	138
Zanoletti	2020	RCS	100
Ren	2021	RCS	151
Cianfrone	2022	RCS	26

Abbreviations: n.a. not available; RCS retrospective cohort study; PCS prospective cohort study; NR narrative review.

**Table 3:** Prognostic factors available and their role in predicting hearing outcome in HPS. SNHL: sensorineural hearing loss.

Prognostic Factors	Studies Describing a Significative Prognostic Role	Studies Not Finding a Significative Prognostic Role
Tumor size	Kemink 1990, Fischer 1992, Nadol 1992, Cohen 1993, Robinette 1997, Mohr 2005, Gjuric 2007, Jacob 2007, Tringali 2010, Sughrue 2010, Mazzoni 2011, Ansari 2012, Sun 2018, Zanoletti 2020, Ren 2021	Goddard 2010, Rachinger 2011, De Freitas 2012, Lovato 2019, Huo 2019
Tumor growth rate	Lovato 2019	Gjuric 2007
Preoperative Hearing	Kemink 1990, Fischer 1992, Nadol 1992, Cohen 1993, Robinette 1997, Tringali 2010, Rachinger 2011, Mazzoni 2011, Di Maio 2011, Lovato 2019, Ochal-choińska 2019, Huo 2019, Zanoletti 2020	Mohr 2005, Gjuric 2007, Jacob 2007, Goddard 2010, Sun 2018
Tumor origin	Cohen 1993, Jacob 2007, Goddard 2010, Rachinger 2011, Huo 2019	Gjuric 2007, Lovato 2019
Surgical approach	Kemink 1990, Sughrue 2010, Nguyen 2012, Ansari 2012, Mazzoni 2018, Ren 2021,	Noudel 2009, Sameshima 2010, Rachinger 2011, De Freitas 2012
Radiological characteristics	Kocaoglu 2003, Tringali 2010, Goddard 2010, Nguyen 2012, Tu 2019	Selesnick 2001, Gjuric 2007, Sun 2018, Huo 2019
Preoperative neurophysio-logical tests	Robinette 1997, Di Maio 2011, Sun 2018, Hochet 2018, Ochal-choińska 2019, Zanoletti 2020, Cianfrone 2022	Kemink 1990, Fischer 1992, Nadol 1992, Cohen 1993 Gjuric 2007, Jacob 2007
Symptoms (other than SNHL)	Huo 2019, Zanoletti 2020, Ren 2021	Lovato 2019
Demographics features	Ren 2021	Fischer 1992, Nadol 1992, Cohen 1993, Robinette 1997 Sughrue 2010, Rachinger 2011, Sun 2018, Lovato 2019, Zanoletti 2020

## Data Availability

Not applicable.
